# Teachers' perceptions of Brandon's Matrix as a framework for the teaching and assessment of scientific methods in school science

**DOI:** 10.1007/s11165-022-10044-y

**Published:** 2022-03-26

**Authors:** Alison Cullinane, Judith Hillier, Ann Childs, Sibel Erduran

**Affiliations:** 1 Department of Education, University of Oxford, 15 Norham Gardens, Oxford, OX2 6PY UK; 2 The Norwegian Centre for Science Education, University of Oslo, Oslo, Norway

**Keywords:** Practical science, High stakes assessment, Methods in science, Brandon’s Matrix

## Abstract

This article utilizes a framework for classifying different scientific methods suggested by a philosopher of science (Brandon *Synthese*, 99, 59–73, 1994) called Brandon’s Matrix. It presents findings from teachers who took part in a funded project in England that looked at the nature of scientific methods in science investigations. Science investigations are an integral aspect of science education and, as such, are often included in high stakes examinations. Therefore, teachers need to have a good understanding of  a range of scientific methods and their purposes in science investigations. The framework was used to ask teachers to classify science investigations based on how they teach them. It was also employed to devise assessments to measure students’ understanding of scientific methods. The teachers were introduced to the new approaches and their perceptions were gathered to understand if they supported this as a framework for their classroom practice. Evidence from the study suggested that Brandon’s Matrix appealed to teachers as a framework for practical science in schools, and they see potential benefits for its use in the teaching, learning, and assessment of science. Findings from the study showed it appealed to the teachers as a tool for classifying scientific methods, and how they also recognized the importance of assessing practical work and had an appreciation of the constraints and drivers in the current curriculum and assessment requirements in England. Implications for teachers’ professional development are discussed.

## Introduction

The article presents findings from teachers who took part in a funded project in England that examined the nature of scientific methods in science investigations. The paper highlights the importance of developing teachers’ understanding of scientific methods by using a novel theoretical framework called Brandon’s Matrix (Brandon, 1994). Science investigations are an integral aspect of science education and, as such, are often included in high stakes examinations (Cheng & Curtis, 2004; Creese et al., 2016). Therefore, teachers need to have a good understanding of scientific methods and their purposes in science investigations. Science as a discipline is often governed and directed by a number of methodological approaches (Irzik & Nola, 2014) that include constructing hypothesises that are testable, avoiding ad hoc changes to theories, choosing a more explanatory theory, rejecting inconsistent theories, accepting a new theory only if it can explain the successes of its predecessor, using controlled experiments to test casual hypotheses and using blinded procedures when conductiong experiments involving human participants (Erduran & Dagher, 2014). Understanding this range of scientific approaches can be considered a key component of scientific literacy – a skill highly valued in science education (Reiss et al., 2012). However, these well-known approaches in science are rarely discussed in school science. Textbooks often feature an over-simplified version of scientific methods that presents science in a linear stepwise fashion called ‘the scientific method’ which is usually associated with manipulative hypothesis testing. This depiction of ‘the scientific method’ in many textbooks has dominated imagery of science for decades (McComas, 2008), despite the notion of it being the only way to conduct science having been widely contested for the past 20 years, due to its skewed representation of science as formulaic and its false view of how science actually works. As a result, this image can damage the reputation of branches of science that do not utilize this stepwise approach (Lederman et al., 2002). When science is not conducted in such a way, it can often be overlooked as unscientific and can lead to mistrust in science (Eichengreen et al., 2021). Representations of the scientific method vary from three to eleven steps, on average depicting five to seven steps. Those steps usually include observing, making hypotheses, experimenting, analysing data, confirming or rejecting the hypotheses and drawing conclusions. It is perceived to serve as a useful package for scientific inquiry and serves as an easy way of seeing science as an organized activity that is ruled by a number of methods (Irzik & Nola, 2014, p1003). Irzik and Nola (2014) noted that methodological rules are often overlooked in school science but are heavily examined by philosophers of science, and it is well-documented that what is taught in school science is not only influenced by the curriculum and associated textbooks but also by the content and style of the assessment, particularly in a high-stakes external assessment context such as England where currently all laboratory work is indirectly assessed through written summative examinations.

This article utilizes a new framework for classifying different scientific methods suggested by one such philosopher of science (Brandon, 1994). The framework was used to devise assessments to measure students’ understanding of scientific methods, but any such introduction of new approaches needs teachers to understand and support this as a framework for their classroom practice (Banner et al., 2012; Tracy, 2014). Hence, this paper will report on a baseline study about teachers’ views of these assessments, thus contributing to the research literature on teachers’ views about the teaching and assessment of scientific methods and investigative work. While we recognize that other jurisdictions may vary in their curriculums and topics taught and assessed, many do include laboratory investigations in conjunction with a system of high-stakes examinations and many test science investigations in their written high-stakes examination papers with these high-stakes tests driving much of what is taught in the classroom (Cullinane & Liston, 2016; Erduran et al., 2020; Creese et al., 2016). Terminology related to investigations in science varies from one jurisdiction to another which can be confusing when engaging with literature on this topic. For example, the terms used include experimental work, practical work, practical and inquiry investigations and skills, practical and investigative activities as well as science investigations (Erduran et al., 2020, Navy et al., 2021), practical experimentation, practical scientific methods (Department for Education, 2014) and modes of inquiry. In the UK context, the term practical science is used most frequently to discuss these laboratory-based investigations in school and will be the term used hereafter to describe the school-based activities in this paper.

## Literature Review

Having identified some of the problems with presenting an over-simplified ‘scientific method’, the literature will be discussed with regard to the need for a diversity of scientific methods to be integral to school science and some of the frameworks that might support this pedagogical aim. Brandon’s Matrix will be introduced and discussed as a framework which does represent a diversity of scientific methods, and the influence of the high-stakes assessment on teaching and learning about scientific methods will be explored further.

### The Importance of the Diversity of Scientific Methods

The depiction of ‘the scientific method’ in many textbooks has dominated imagery of science for decades (McComas, 2020, Reiff-Cox, 2020, Emden, 2021). The introduction outlines the prevailing presentation of science as having a single scientific method, which does not reflect the diversity of approaches used by scientists. For instance, astronomers cannot manipulate the planets, black holes or other astral bodies they study. A chemist may perform a titration to observe an outcome or obtain a measurement only, and Darwin was able to build his theory of evolution by natural section through observations of a variety of species over time (McComas, 2020). When one examines scientific papers, they seem to present manipulative hypothesis testing as ‘the’ scientific method. In reality, practising scientists use a variety of approaches to solve a problem, formulate hypotheses or make relationships in the data. Research scientists will know that published papers are reconstructed accounts of completed work. They were composed to fit a standardized publication format. They do not describe how research occurs in practice in laboratories, universities and in the field (Wivagg & Allchin, 2002). These professional accounts of science in action are not an accurate portrayal of how science works. The problem with these accounts is that students do not see the uncertainty, incompleteness or modifications that forms part of the process. They are excluded from the process and students come to believe that the ‘scientific method’ guarantees discovery, unequivocal and reliable conclusions (Wivagg & Allchin, 2002). School science does not help to dispel this myth when the school textbooks also depict a sanitized version of the scientific process and a presentation of science theory with little understanding of how it came to be science knowledge (Wei et al., 2021). Science investigations in school do not help matters either, where students are frequently presented with a set of steps to follow like a recipe. This often leaves students feeling dismayed when they did not get *the* right data and are subsequently supplied with *the* right data so that they can reproduce it in a test or analyse it in high-stakes examinations (Abrahams & Millar, 2008).

There have been some attempts to represent a broader range of scientific methods by frameworks opposed to ‘the scientific method’ (e.g. Lawson, 2003; Rudolph, 2005; Turner, 2013). One such approach has been put forward by Reiff-Cox (2020). To combat the linear approach, Reiff-Cox (2020) developed the inquiry wheel which she proposed as a model to show diverse pathways in science and is ‘a dynamic representation of scientific processes, which continues as long as questions both large and small continue to fuel the investigation’, (p.130). The development of the wheel came from distilling activities stated by 52 scientists from biology, environmental science, chemistry, medical sciences, physics and geology about their approaches and conceptions of scientific inquiry during interviews with them. The inquiry wheel model depicts scientists generating questions along each stage and revisiting previous stages whenever needed. Reiff-Cox (2020) outlines how these questions and their answers are the figurative force necessary to turn the wheel for an investigation to proceed (p.131). The stages of the wheel include (1) observing, (2) defining the problem, (3) forming the question, (4) investigating the known, (5) articulating the expectation, (6) carrying out the study, (7) interpreting the results, (8) reflecting on the findings, and (9) communicating the findings to the scientific community and society. An alternative framework was proposed by Sturdivant-West et al. (2020) as an illustration of the ways that investigations can occur in science. It presents a detailed overview of scientific methodology in what they describe as a useful tool called the ‘Modes of Scientific Inquiry’ (MSI) flowchart. The flowchart would be a useful addition to the science classroom as it provides examples of qualitative and quantitative methods, observational attributes, useful investigative approaches with potential graphical representations of results from such analytical investigations; in short, a useful summary of ways to conduct authentic scientific research. While the inquiry wheel and the MSI flowchart are useful to see the full range of activities utilized by scientists, the images are less helpful as a pedagogical tool for classroom practical activities to help students understand what it is that they are doing when they are doing it (McComas, 1998). Neither are they mapped to current curriculum or assessment demands in the UK, making them more challenging for teachers to use in the classroom. The study reported here used a framework that not only reflected the diversity of science methods but was also accessible to teachers as a teaching tool and to students as a learning tool during practical investigations.

### Introducing Brandon’s Matrix

Brandon’ Matrix (1994, p.64) presents an alternative approach to ‘the scientific method’ through a matrix (see Table 1), recognizing that there is no single scientific method, but instead scientists utilize a range of methodological approaches and that these different approaches are essential. Brandon (1994) describes the nature of a practical investigation, which he identifies as an experiment and/or an observation, and by whether it involves the manipulation of variables, testing of hypotheses or simply measuring parameters. Through his two-by-two matrix, Brandon (1994) illustrates how not all investigations in science rely on hypothesis testing and not all observational work is non-manipulative (Brandon, 1994; Emden, 2021).

**Table 1:** Types of scientific methods (reproduced from Brandon, 1994, p.63)

Experiment/observation		
	Manipulate	Not manipulate
Test hypothesis	Manipulative hypothesis test	Non-manipulative hypothesis test
Measure parameter	Manipulative description or measure	Non-manipulative description or measure

Brandon’s Matrix provides a framework to aid understanding of methods used in scientific investigations, and how the findings from a range of different methods can be used to explain different scientific concepts and phenomena. It provides a *hands-on, minds-on* approach to scientific methods and helps students see the purpose of different approaches to laboratory work and can be used as a way to classify classic or historical experiments presented in textbooks. Depending on the learning aims, the same investigation can be carried out using different approaches and can be categorized in multiple quadrants of the matrix. An example for chromatography is provided in Table 2. We acknowledge that not all investigations can be carried out in all four quadrants of the matrix, but as a widely used technique, chromatography is a useful example to demonstrate how it can fit into all four quadrants of the matrix and be taught and presented in a variety of ways (Erduran & Wooding, 2021).

**Table 2:** Chromatography investigations which could be used in science lessons to *investigate the colours in a sample of purple ink* as presented through Brandon’s Matrix

Experiment/observation *Using paper chromatography, investigate the colours in a sample of purple ink*		
	Manipulate	Not manipulate
Test hypothesis	**Hypothesis:** All purple inks are composed of blue and red pigments **Variables:** Independent variable: different purple inks Dependent variable: the pattern of separation	**Hypothesis:** Purple ink is a mixture of red and blue inks Variables: No manipulation of variables
Measure parameter	Measure the R_f_ values of a range of inks **Variables:** Three different inks	Categorize a range of inks into ‘pure’ pigments and mixtures

As well as contemporary classroom practicals, the matrix was used to classify well-known historical examples of scientific investigations which are often presented in the science classroom to illustrate the development of scientific knowledge (see Table 3). They clearly show how science does not fall into examples of the single ‘scientific method’, as the table nicely reflects the multiple approaches in Brandon’s Matrix (Wooding et al., 2020). These examples were used with the teachers in the initial meeting and were successful in helping them understand the matrix in more context.

**Table 3:** Historical investigations often used in school classrooms as categorized by using Brandon’s Matrix

Experiment/observation *Historical examples that used different scientific methods*		
	Manipulate	Not manipulate
Test hypothesis	**Hypothesis:** Louis Pasteur’s experiments to test the hypothesis of ‘spontaneous generation’ **Variables:** a broth that was exposed to a source of microbial cells, and a broth that was not.	**Hypothesis:** Foucault observed a simple pendulum to support his hypothesis that the Earth was spinning and not that the universe was spinning around the Earth.
Measure parameter	Eratosthenes’ measurements of the proportions of the Earth to calculate its circumference. **Variables:** Measuring the shadow of a stick in two different locations during the summer solstice and using geometry calculations.	Mendeleev’s construction of the prototype periodic table of elements where he placed the elements in rows by increasing atomic mass. He was able to predict future elements yet to be discovered.

One study conducted by Cullinane et al. (2019) used the matrix as an analytical framework to examine how scientific methods are framed in high-stakes science examination papers. The findings of the paper illustrated how manipulative parameter measurement dominated chemistry examination questions, and how manipulative hypothesis testing type questions were present in a limited capacity. Such observations are contrary to expectations that manipulative hypothesis testing would be dominant, as this is often presented as ‘the scientific method’ in many science lessons around the world. Brandon’s Matrix is gaining traction internationally where it was recently used to examine practical investigations in textbooks in China (Wei et al., 2021). This analysis was conducted on practical work compiled in nine textbooks of biology, chemistry and physics used in the stage of junior high school (grades 7–9). Similar to the findings by Cullinane et al. (2019), Wei et al.’s (2021) study found that non-manipulative parameter measurement (NPM) was the dominant category identified in physics, chemistry and biology textbooks. These studies show the disconnect between what is presented as methods in school science, such as the methods students are performing to draw conclusions from investigations, and the methods tested in textbooks and high-stakes examinations.

Brandon’s Matrix has the potential to offer a simple framework for categorizing scientific investigations that reflect a more balanced image of the diversity of scientific methods. Our work (e.g. Ioannidou & Erduran, 2021) has begun to investigate teachers’ views of the matrix as a framework for teaching and assessment of scientific investigations. Previous innovations in curricula and assessment have not always been successful if teachers have found them to be cumbersome or lacking in relevance (Hillier, 2012). However, Brandon's Matrix has been effectively introduced introduced in pre-service teacher education (Kaya et al., 2019), the intention was to use Brandon’s Matrix as a framework for the development of new summative assessment questions that would include a wider spectrum of scientific methods. For this purpose, a group of trained examiners developed a set of six assessments for General Certificate for Secondary Education (GCSE) level (students aged 14–16 years old) on the topics of osmosis, ecology, electromagnetic spectrum, circuits, chromatography, separation and mixtures (Project Calibrate examination papers, 2020). It was important that science teachers were also part of this initiative and that their views, reported here, were taken into account in the development process, as teachers’ understanding and supporting this move would be crucial for any subsequent policy change to be effective (Banner, Donnelly & Ryder, 2012; Tracy, 2014).

### What Influences the Teaching and Learning of Scientific Methods?

Before describing the study further, it is important to justify this focus on high-stakes assessment. High stakes assessment is one of the most influential drivers of what is taught and learned in the classroom (Creese et al., 2016). Literature reports that it can have a negative backwash effect with teachers only ‘teaching to the test’ which can distort the teaching and learning process in unhelpful ways (Cheng & Curtis, 2004; Popham, 2001; Volante, 2004). For example, Volante (2004) reports that in the North American context, high stakes testing has had the effect of focusing teaching more on knowledge acquisition rather than, for example, developing students’ ability to apply knowledge ‘in a novel situation’ (p.1). This is in agreement with a study in the Republic of Ireland that looked at high-stakes biology assessment, which found that almost 90% of the questions in 20 years of examination papers fell into lower recall type questions, supporting this link between high stakes assessment and low-level knowledge acquisition (Cullinane & Liston, 2016). In England, the assessment of laboratory investigations moved from being classroom-based to 100% written high-stakes assessment for students age 16 in 2015, with the General Certificate for Secondary Education (GCSE) specifications typically being taught to 14–16-year-olds (DfE, 2014; Cramman et al., 2019). The curriculum set by the Department for Education (DfE) requires students to complete a minimum of eight to ten hands-on practicals, but schools are unrestricted in the amount of practical work they can undertake (Ofqual, 2015). The examination awarding bodies have prepared documents with suggested methods for carrying out the required practical activities and give ideas and guidance to help teachers plan the best experience for their students. These methodological approaches are not compulsory and the teacher is free to plan alternative approaches to conduct the investigations (see, AQA, 2018b; AQA, 2018c; AQA, 2018a). Questions on the written examinations focus on these specified ‘hands-on’ practicals or variations of these, in the high stakes written examinations taken by students aged 16 years old. Cadwallader (2019) outlines how the intention was to facilitate more frequent practical work that integrated curricular content better and would be assessed in a valid and manageable way. However, this indirect assessment of practical science skills is contentious as it is thought that written examinations do not fully test the skills necessary to bring together the ‘hands-on’ and the ‘minds-on’ to understand the underlying scientific principles (Abrahams et al., 2013; Harlen & James, 1997). Research findings on high-stakes science and mathematics assessments indicate that they can be damaging, as often they can indirectly reduce the quality of teaching and learning, as teachers focus on teaching what they know will be tested, narrowing the curriculum which results in narrowed practical skills set (Erduran et al., 2020). The washback effect of high-stakes testing reduces autonomy for teachers who might be reluctant to experiment with new pedagogies due to time and accountability constraints (Klenowski & Wyatt-Smith, 2012; Skipp & Dommett, 2021). Concerns around this transition led to the development of this project to design practical assessments that would engage the *hands-on* with the *minds-on,* using Brandon’s Matrix as a framework to support the development of these assessments. As indicated earlier, it was important that science teachers were part of this process, and hence a case-study methodology was implemented to capture in-depth insights into the teachers’ perceptions of Brandon’s Matrix. The purpose was to find out their views about whether they feel Brandon’s Matrix was an appropriate framework to use for the assessment, teaching and learning of practical work. In order to do this, we first decided to get their current perceptions of practical science and assessment in practical science because we recognised that these existing views would affect the way they interacted with Brandon’s Matrix and what it offers as a framework. If they had strong views supporting 'the scientific method', they might find the diversity of the matrix difficult to accept. Conversely, if they found the current assessment practices constraining, they might relish the opportunities presented by the Matrix or it still may not be in alignment with their views about practical work.

Therefore, our first two research questions are (1) What are teachers’ perceptions of practical science? (2) What are teachers’ perceptions of assessment of practical science?

The following overarching research question guided the study: (3) What are teachers’ perceptions of Brandon’s Matrix as a framework for describing the range of scientific methods being taught and assessed in school science?

## Methodology

The study took the form of an ethnomethodological study to evidence the potential of Brandon’s Matrix as a framework for the assessment of practical science in high-stakes external written examinations in everyday teaching (Barker 1997). The assessment of practical science and associated skills is a contemporary issue (OECD, 2019), and the case-study approach was appropriate as this needed to be understood within the real-world context of current GCSE examinations. This also had other key features typical of a case-study design, including using evidence from multiple teachers, with a number of variables of interest, all underpinned by existing theoretical ideas about the importance of practical work to the learning of science, the challenges of assessing practical work, and Brandon’s Matrix itself as a framework reflecting the diversity of scientific methods (Yin, 2018). The study will be briefly outlined, followed by a description of the participants and the methods of data collection and analysis.

### Participants

The six teachers who attended the professional development were recruited through a convenience sampling method via local teacher networks and they all taught in partnership schools who work with the University’s teacher education programme. The teachers, who voluntarily participated in the study, had a mixture of subject specialisms to ensure cross-subject representation. They all taught in state schools and were from five different schools, as shown in Table 4. Each teacher taught a mixture of science subjects to students 11–16 years of age (lower & middle school) and taught their own specialism to students aged 16–18 years old (high school). At the onset of the project, ethical clearance was granted by the University’s ethics committee and all teachers involved provided their informed consent to record their data.

**Table 4:** Science teachers’ background and experience at the time of the study

Teacher	Subject specialism	Teaching experience	Gender	School type -all were state schools
*Teacher 1*	*Physics*	*23 years*	*Female*	*Suburban*
*Teacher 2*	*Biology/Physics*	*9*	*Female*	*Urban*
*Teacher 3*	*Biology*	*5*	*Female*	*Suburban*
*Teacher 4*	*Chemistry*	*6*	*Male*	*In the same urban school as Teacher 5*
*Teacher 5*	*Biology*	*8*	*Male*	*In the same urban school as Teacher 4*
*Teacher 6*	*Physics*	*5*	*Female*	*Suburban*

### Study Outline

Brandon’s Matrix was being applied in the three-year funded Project Calibrate (Cullinane et al., 2019; El Masri, Erduran & Ioannidou, 2021; Erduran et al., 2021; Erduran & Wooding, 2021; Ioannidou, Finch & Erduran, 2022; Ioannidou & Erduran, 2021) to support the teaching and learning and assessment of practical science. Project information and resources are available at the following link: https://ProjectCalibrate.web.ox.ac.uk/. For the purposes of this paper, we wanted to gather teachers’ views of Brandon's Matrix for supporting students' understanding of scientific methods in the English context. The matrix was previously used in other studies in Turkey for professional development purposes (Kaya et al., 2019). The first stage was to bring together experienced science teachers in a professional development meeting where they were introduced to the matrix and provided with an illustrated example of the matrix in relation to historical investigations, and the reasoning behind using it for pedagogical practice. To help the teachers see the utility of the matrix, they were asked to use the framework as a classification tool. The teachers were supplied with cards for practicals on the General Certificate of Secondary Education (GCSE) curriculum, and directed to place the cards into a quadrant in Brandon’s Matrix based on how they would usually teach each practical. Subsequently, the teachers were introduced to the assessments that had been designed for the study. The assessment questions were in the style of GCSE questions and were designed with a team of examination writers from one of the leading examination awarding bodies in England who used Brandon’s Matrix as the theoretical framework in the development of the questions. Each quadrant of the matrix was represented in each set of questions for six topics across the three science subjects (two biology topics, two chemistry topics and two physics topics). After the meeting, the teachers were asked to participate in an interview about their views on Brandon’s Matrix, how practical science contributes to students’ learning of science, and the assessment of practical science.

### Teacher Interviews

Following the meeting, the teachers were interviewed, with these being audio-recorded and transcribed. The interview questions were developed to elicit teachers’ views about practical science, the assessment of practical science and their views on Brandon’s Matrix. Although the focus of the study was their views on Brandon’s Matrix, it was recognized that these were likely to be heavily influenced by their existing views on the role of practical work in learning science, and on the assessment of practical work; hence these aspects were explored with the teachers first, before moving onto discussion of Brandon’s Matrix itself. The ethical procedures were outlined to the teachers; they would remain anonymous and any information they gave would be treated with the strictest confidence, and no outcomes would be reported that could identify either their school or them as individuals.

### Coding and Analysis

The interview data were analysed through thematic analysis, an approach commonly used for capturing patterns across qualitative datasets. The coding methodology implemented a deductive coding process where themes emerged from participants’ spoken word (Fereday & Muir-Cochrane, 2006; Saldaña, 2009). The analysis sought to provide insight into teachers interpretations to afford direct representation of these individuals’ viewpoints and descriptions of experiences, beliefs and perceptions of Brandon’s Matrix, and what the teachers said about practical science and their views on the recent changes in assessment (Fereday & Muir-Cochrane, 2006). Initially, two of the authors coded one transcript together to develop a coding frame which resulted in consensus agreement on the codes. Then two more transcripts were independently coded by each rater and subsequently co-coded. This process was repeated for the remaining transcripts until the codes agreed. This coding frame was then given to a third reviewer to act as an independent rater, until the inter-rater agreement was higher than 95% (Elliott, 2018).

## Results

Before the teachers were asked about their views of Brandon’s Matrix, the interview questions sought their views on practical science, as this had recently undergone a reform close to the time of the study. They were asked about their views on the assessment of practical science, as this formed a large part of the reform efforts where the assessment of practical science now took place through written examinations, and of course they were asked their views on practical science, as any comments on Brandon’s Matrix would need to be interpreted in the light of their views on the real-world context of practical science and its assessment in relation to the school system.

### Teachers’ Perceptions about Practical Science

The interviews revealed that the teachers’ views about practical science centred around two themes: the teaching and learning of practical science, and the nature of the discipline or subject. In relation to teaching and learning, the findings show that the teachers viewed practical science as fundamental to the definition of science and to the development of students’ practical skills. They viewed practical science as motivational, increasing enjoyment of the subject for both students and teachers. The teachers also viewed practical science as a key pedagogical tool to make the abstract ideas of science more accessible to students as well as communicating that there are investigative processes in science and findings in science are based on evidence from data produced by different types of experiments and scientific observations. When talking about practical work as fundamental to science and to the learning of science, these quotes below were indicative of the teachers’ views.

*‘It defines what science is, it underpins everything. It is the ability to test, it is the ability to use data and results to inform your thinking. Although there are many examples of thought experiments and things like that which are perfectly valid, at the GCSE level it’s hands-on with equipment, it’s learning the skills as well as learning how to interpret.’* (Teacher 1)

[Practical science] ‘*communicates that the process is there and what it is that is around them. There is so much fake news around now. It helps to show them there is a process and science is based on evidence*’ (Teacher 5).

The teachers often talked about the variety of skills required to plan and carry out practical work and investigations, indicating how practical science was used to develop students’ proficiency in these skills. Furthermore, they also viewed practical work in the form of investigations as involving the need to develop multiple practical skills, and therefore they perceived the need to give their students plenty of practice in carrying out investigations:

*‘It’s the ability to test, it is the ability to use data and results to inform your thinking... there are nuances to interpreting data.... the planning and the understanding of designing an experiment is so crucial. What are your parameters? What are you actually looking at?’* (Teacher 1)

*‘There is quite a lot of data analysis... graph skills and those sorts of things, so I do focus on that a lot to make sure that the kids can [..] make the graph, interpret the graph*.’ (Teacher 3)

Teachers discussed how they and their students enjoyed doing practical work, and so practical work was a useful motivation tool for the students. One teacher discussed their own enjoyment of the practical element and how practical science is a motivation for being a science teacher.

*‘For me it’s for motivation and it’s the part that I enjoy the most. It’s a bit selfish but I enjoy it, I like doing it. Students are having a good time as well, it’s a big part of why I like being a teacher.’* (Teacher 4)

*‘As you know, there is a lot of content I need to get through. If I have the luxury of time I will do as much practical work as possible. I love doing practical work and want to incorporate it as much as possible. If I run out of time I will do less.’ (Teacher 5)*

Such was their view of the value of science both to build practical skills and to motivate students, teachers in this study said that they did not feel constrained by the curriculum requirement (i.e. to do just the eight required practicals) and reported that they took every opportunity to include practical work in their teaching.

*‘I don’t limit to the required practicals at all. Where at all possible, I will try and fit a practical in*.’ (Teacher 4)

In addition, the teachers perceived that some students find learning science difficult due to its abstract nature. They talked about the difficulty many students have linking theory to practical work or, conversely, linking the practical to the theory for the examination:

*‘We did chromatography about three lessons the other week, “we can do this,” but as soon as I’m like, you know, let’s do some theory work, they kind of fold in themselves, “Miss, I can’t do this, I can do chromatography, I can tell you exactly how to do the practical, I can tell you why we do the line in pencil” They’re really excited about that because they can do it correctly, but as soon as I’m like “Right let’s do an exam style question about that” they’re like “Oh no, we can’t do that”.’* (Teacher 3)

However, despite these challenges, the teachers also viewed practical work as an opportunity to demonstrate abstract theory in practical applications which helps to consolidate learning by making science more understandable. One teacher noted how *‘It makes science a bit more real.’* (Teacher 2).

Another commented: *‘It helps them with their understanding as to where all the ideas that we are teaching them comes from. We can teach them what it means, but once they actually do it themselves, it becomes clearer in their minds.’* (Teacher 3)

Finally, the teachers viewed practical work as a key vehicle to relate the school subject of science to how scientists work (nature of science), drawing out the parallels between school practical science and the work of scientists. One teacher took this view further and related this to career relevance in science and what they do in class to promote careers and many emphasized they felt practical science promoted the life skills of problem solving.

*‘It’s the scientific skills that students develop when they actually do practical work and, its usually the things that they, you know if they want to go into sciences as a career choice, that’s what they usually enjoy most about this, it’s the sort of scientific thinking and the scientific discovery process.’* (Teacher 2)

*‘Not everyone is going to be a scientist, but everyone needs to problem solve. If they can link a question to evidence, [practical science] will help them as they go along’* (Teacher 6)

The findings above demonstrate that the teachers clearly recognize the multifaceted nature of practical science and the ways it can be used to engage and support students in their learning of science.

### Teachers’ Perceptions of the Assessment of Practical Science

Having explored the teachers’ views about practical science, they were asked about their views on the assessment of practical science. Analysis of the data showed teachers expressing some views about the advantages of the current system of assessment but they also had a number of concerns. Previously, the assessment of practical science had been through ‘controlled assessments’; practical investigations carried out and written up by students in school laboratories under examination-like conditions and graded by their teachers. Firstly, they recognized the advantages and ease of testing large numbers of students in external written examinations which are graded by external markers; these were clearly logistically much easier for schools than conducting multiple experiments in lessons with all the associated demands on teachers, both to set up and manage these and also to carry out the assessment. Additionally, the teachers recognized that the return to written examinations mitigated against the challenges associated with high-stakes testing where they talked openly about the ‘gaming’ of the system through excessive coaching of students and also by the fixing used by some schools and teachers to improve students’ grades:

*‘The coursework was used to bump up the grades’*. (Teacher 1)

*‘I do know some schools were quite guilty of coaching towards those.’* (Teacher 3)

*‘Always with any sort of exams, you try and find the cheat codes.’* (Teacher 4)

The teachers also perceived a number of disadvantages with the current assessment of practical work at GCSE. Firstly, they perceived that the current written examinations offered very limited opportunities to assess students’ practical skills and their understanding of the scientific process:

*I think what it (written assessment) doesn’t do is test the skills....but it doesn’t test the scientific process, it doesn’t test the real lab skills*. (Teacher 2)

As a result, when asked how they would like to see practical science be assessed in the future, they expressed the need for a mixture of approaches to assess the full range of skills developed through practical work:

*‘I think maybe a combination of how it is done now with the questions in the written exams plus something about how the skills…you can’t really assess skills unless they’re actually doing the practical* …[….]… *I think what it [written assessment] doesn’t do is test the skills....but it doesn’t test the scientific process, it doesn’t test the real lab skills.’* (Teacher 5)

In addition, when talking about the transitions between GCSE and A levels (external examinations taken by students aged 18 years), the teachers identified that current assessment procedures at GSCE leave students ill-prepared for the planning and inquiry skills necessary at A-level.

*‘I think they were very “led” even though it was supposed to be independent.’* (Teacher 6)

*‘The very first practicals we do at A-levels are totally recipe following, ... I expect more from my pupils. I expect error analysis...I want [them] to plan.... I get slightly the feel that they’re not building on what we built in at GCSE. ... We need to step it up a little bit.’* (Teacher 1)

*‘I don’t think they come out of the GCSEs thinking that science is a practical subject.’* (Teacher 2)

The final challenge was about the assessment criteria for practical work as outlined in the curriculum, and in particular, how to resolve the tension between the types of questions typically asked in written examinations and the ability of these questions to check that students had genuinely carried out the practicals in class. They perceived that the questions on the current written examination led students to rote learn the procedures and they also perceived that the questions were not able to discriminate between those students who had carried out the practicals and those who had not.

*They’re so focused on rote learning because of the sort of specifications we now have.* (Teacher 2)

*Are they asking the right questions? How do we make sure that the students have actually physically done the practical? That’s the problem*. (Teacher 1)

The findings above suggest that the teachers have a nuanced understanding of the challenges and complexities of assessing practical science in a valid and meaningful way.

### Teachers’ Perceptions about Brandon’s Matrix

At the initial meeting where the teachers were introduced to Brandon’s Matrix, the teachers were asked to place the cards with practical investigations into a quadrant in Brandon’s Matrix based on how they would teach these. This allowed discussion for the reasons for the placement of the cards. The ensuring discussions yielded some interesting insights into their framing of the practicals, according to the teachers’ own subject backgrounds. It appeared that the teachers were less likely to present the practical as a hypothesis test if they were teaching outside their own subject specialism (e.g. a biology teacher teaching physics, for example). In the interviews, the teachers were asked about their views of Brandon’s Matrix as a framework for practical science. Analysis of the data categorized their views into two main themes; the first theme included comments focused around the positive uses for Brandon’s Matrix which the teachers had identified for the teaching and assessment of practical science. The second theme focused on some concerns about its suitability for use by teachers and students, alongside some suggestions to better support the use of Brandon’s Matrix in the classroom. The teachers indicated that there were a number of ways in which Brandon’s Matrix could be used that would be beneficial for practical science. The first was as a meta-tool for the teachers to visualize how they teach and present practical science. The teachers saw it as a useful organizational tool for the current practical activities in the curriculum, and that using it also demonstrated how current practical activities are lacking in some methodological areas.

‘*I think it is a really good way [...] of like organizing the practicals that we already have within our curriculum. It makes it really clear to see what we […] assess and what we focus on a lot, and where our areas of weakness are. 110%. As soon as we laid it all out, I was like wow, just look at it, it’s so clear what’s lacking and what isn’t. I thought it was a really good model, really clear model and quite easy to understand’* (Teacher 3)

Second, teachers felt it could be used with students to afford them the opportunity to think and reflect more about the practical activities because it aligns more closely with what practical work should do, as current procedures are failing to do this.

*‘I like the idea of making them think about the experiment, which is what it did. It made them think “does it do that or doesn’t it?” and I like that because that’s what we don’t do.’* (Teacher 1)

Third, they also felt it was a useful framework to present a broader view of practical science beyond what is typically presented and hence could foster students’ interest in science. When asked if they thought Brandon’s Matrix was worthwhile to include in their teaching of practical science, a teacher stated that the framework provides a particular realization for the students in terms of providing an alternative view of science:

*‘It opens up a path to science that otherwise children will overlook or will be more likely to overlook because they don’t realize that it’s a part of the scientific discovery process.’* (Teacher 2)

Finally, some teachers also recognized the simplicity of the framework and their own ease of understanding. They felt it was clear and easy to understand, particularly with the help of the activity that was carried out in the meeting.

‘*I think it is a really good way of kind of like organising the practicals that we already have within our curriculum it makes it really really clear to see what we clearly do assess what we focus on a lot where areas of weakness are. 110%. I thought it was a really good model, really clear model and quite easy to understand as well.* ’ (Teacher 3)

‘*It’s a nice flow diagram, very clear, it’s a way of categorizing practical methods*.’ (Teacher 4)

Although there was a largely positive reaction to the use of Brandon’s Matrix, some teachers voiced potential concerns about the accessibility of the model and that it might need further translation for both teachers and students. To allow the matrix to become embedded in teaching culture, they perceived training would be needed in how to use the matrix and understand it. They also perceived that teachers would be critical players in the enactment of ideas from Brandon’s Matrix and professional development would be helpful to ensure a good level of understanding of the matrix as a framework for teaching and assessment. Such observations are, of course, expected of new initiatives that may be unfamiliar to teachers, and professional learning opportunities need to be built into their experiences to ensure that they can adapt new and innovative strategies (Banner et al., 2012). The following teacher’s quote highlights the need for the development of teaching resources for CPD providers from her experience of attending the meeting.

*‘I think you’d probably need CPD training for teachers to do it properly. The A-level training is online, so I think you could probably do it as an online CPD rather than an actual face-to-face thing. I would think [that is] definitely achievable.’* (Teacher 2)

As said, teachers were also concerned about how to present the matrix to students and how the language of the matrix (e.g. manipulative, parameter) might be challenging for students. They suggested that the wording of the matrix be made more accessible for those with literacy issues.

*‘The wording has to be accessible and understandable by the pupils. We cannot say “using this matrix, which one shows this hypothesis is observable or non-observable?” You might as well talk in Swahili*.’ (Teacher 1)

As key stakeholders in the wider project, teachers’ feedback were crucial and this has already informed other areas of the study. As a result of the above comment, a booklet for CPD providers has been developed (Wooding et al., 2020). In summary, teachers clearly recognized the affordances and constraints Brandon’s Matrix offers for the teaching and assessment of practical science and could also offer creative suggestions to make its use even more educative and powerful. Such recommendations are consistent with studies that our team has conducted with a different cohort of teachers (Ioannidou, Finch & Erduran, 2022; Ioannidou & Erduran, 2021b). In light of this feedback from the teachers, the project team did indeed change the wording to the more accessible language of the matrix and developed student-friendly heuristics (Wooding et al., 2020).

## Conclusion and Implications

The study reported on three research questions to investigate teachers’ perceptions of Brandon’s Matrix as a potentially useful framework for the teaching and assessment of scientific methods in school science. Their views on practical science centred on their use of it to motivate students, skills development and present how scientist work. Their perceptions of the assessment of practical work in written tests present mixed views about the advantages and fairness of the current system of high stakes examinations but also recognized the limited opportunities written examinations present to develop practical skills. The empirical study unpacks the nature of practical science by appealing to a theoretical framework about the diversity of scientific methods based on Brandon’s Matrix (Brandon, 1994). Having introduced science teachers to Brandon’s Matrix, the teachers’ views about this framework was explored with the intention of identifying how procedural and hands-on aspects of practical work can be meaningfully aligned with thinking and minds-on skills.

The teachers’ perceptions in this study demonstrates that Brandon’s Matrix provides the possibility of such a synthesis in that it reinforces the articulation of why and how an investigation is done in practical science. For example, it differentiates between different approaches to hypothesis testing and promotes awareness of what is being tested in an investigation. As such, the study illustrates that the science teachers participating in the study viewed Brandon’s Matrix as offering an alternative to broaden understanding, not only about methods in science practical work, but also methodological approaches in assessment designed in such a way as to bring the *hands-on* with the *minds-on* goals of science education (Abrahams et al., 2013). Furthermore, the construct of practical science offered through Brandon’s Matrix also allows pedagogical innovation for teachers’ professional development. Schwab (1962) explains how expertise in teaching requires both knowledge of the content of a domain and knowledge about the epistemology of that domain. Science teachers who have an understanding of why and how practical science contributes to science teaching and learning, will be better able to expedite students’ understanding of the role of practicals in science. Such arguments have also been mirrored by other researchers (e.g. Lampert, 1990; Reiff-Cox, 2020; Shulman, 1987) which emphasize the significance of the disciplinary knowledge in good teaching. The implication, however, is that there may be challenges in how teachers’ learning of epistemic frameworks such as Brandon’s Matrix can be supported when such epistemic features are not widespread in professional development experiences (Ioannidou & Erduran, 2021b).

The literature review has highlighted some shortcomings of other frameworks for the teaching and learning of scientific methods. The teachers had a clear understanding of the role of practical work in the teaching and learning of science, and the complexities of assessing practical science. Considering the limited literature on summative assessment of practical science (Abrahams et al., 2013; Watt, 2013), the paper contributes to the body of research by illustrating what teachers of science identify as the key issues of summative assessment of practical science. The findings suggest how the teachers view the systemic issues in relation to examinations in England and the particular nuances around the testing of skills related to practical science. Although previous studies and reports highlighted the problems related to the teaching and assessment of practical science in England (e.g. Osborne & Dillon, 2008), research articulating science teachers’ views is fairly scarce (Navy et al., 2021, Maeng et al., 2020). In particular, there is limited empirical data on English science teachers’ views about the assessment of practical science as well as its teaching. The study thus contributes to knowledge and understanding of English science teachers’ views on a topic that has been of a major concern in England (Reiss et al., 2012; Watt, 2013).

Understanding teachers’ views of practical science, its teaching and its assessment has implications for the design of curricula as well as examinations, given teachers are the important agents of school reform. A systemic approach to the design, evaluation and refinement of summative assessment ensures that teachers’ views contribute to the vital future of practical science. It is important to consider information about how teachers react to curriculum and assessment reform (Abrahams et al., 2013). If we can inform policy makers about the impact of these curricular changes from the teachers’ perspectives, it can allow teachers to have a say in the potential future policy changes. Research about science teachers’ views is therefore valuable in potentially providing some input into the content of curriculum and assessment policies, given teachers will be at the receiving end of these policies and they will be driving their implementation at the level of the classroom. Our research reported elsewhere (Erduran et al., 2021; El Masri et al., 2021; Project Calibrate Report, 2021c) has also explored students’ views about Brandon’s Matrix. Ultimately the use of a robust construct of practical science in aligning the content and goals of curriculum, instruction and assessment will ensure that there is coherence in the goals and outcomes of science education.
